# Novel and Converging Ways of NOX2 and SOD3 in Trafficking and Redox Signaling in Macrophages

**DOI:** 10.3390/antiox10020172

**Published:** 2021-01-25

**Authors:** Steen Vang Petersen, Nanna Bach Poulsen, Cecilie Linneberg Matthiesen, Frederik Vilhardt

**Affiliations:** 1 Department of Biomedicine, Aarhus University, 8000 Aarhus C, Denmark; SVP@biomed.au.dk (S.V.P.); cl@biomed.au.dk (C.L.M.); 2 Department of Cellular and Molecular Medicine, Copenhagen University, 2200 Copenhagen, Denmark; nannabp@sund.ku.dk

**Keywords:** NOX2, SOD3, Rab27, membrane trafficking, cellular sorting, redox signaling, macrophages, hydrogen peroxide, superoxide

## Abstract

Macrophages and related tissue macrophage populations use the classical NADPH oxidase (NOX2) for the regulated production of superoxide and derived oxidants for pathogen combat and redox signaling. With an emphasis on macrophages, we discuss how sorting into secretory storage vesicles, agonist-responsive membrane trafficking, and segregation into sphingolipid and cholesterol-enriched microdomains (lipid rafts) determine the subcellular distribution and spatial organization of NOX2 and superoxide dismutase-3 (SOD3). We discuss how inflammatory activation of macrophages, in part through small GTPase Rab27A/B regulation of the secretory compartments, mediates the coalescence of these two proteins on the cell surface to deliver a focalized hydrogen peroxide output. In interplay with membrane-embedded oxidant transporters and redox sensitive target proteins, this arrangement allows for the autocrine and paracrine signaling, which govern macrophage activation states and transcriptional programs. By discussing examples of autocrine and paracrine redox signaling, we highlight why formation of spatiotemporal microenvironments where produced superoxide is rapidly converted to hydrogen peroxide and conveyed immediately to reach redox targets in proximal vicinity is required for efficient redox signaling. Finally, we discuss the recent discovery of macrophage-derived exosomes as vehicles of NOX2 holoenzyme export to other cells.

## 1. Introduction

Once thought upon merely as a facet of the microbicidal defense mechanisms of phagocytes, oxidants are now recognized as primary messengers in an intricate and widely arborized network of redox signaling, where the activity of target proteins is regulated by reversible oxidation and reduction cycles of low pKa thiols [1,2]. The cyclic nature of these modifications requires that oxidants are counteracted by antioxidants that maintain an essential redox homeostasis for controlled oxidative reactions.

Since the discovery of the expanded NOX family of superoxide-producing NADPH oxidases in 2000 [3,4], we now know that virtually all cells of our body can produce superoxide in a regulated manner. Phagocytes such as neutrophils with a short life span and a high expression of NOX2 (the classical phagocyte NADPH oxidase [5]) use a range of highly reactive superoxide-derived oxidants to eliminate pathogens. In contrast, long-lived mononuclear phagocytes, such as macrophages and different tissue macrophage populations, express NOX2 at much lower levels; while NOX2-derived oxidants certainly help macrophages combat certain pathogens [6], it is also clear that macrophages and their related tissue macrophage populations use oxidants for both intracellular and intercellular redox signaling purposes [7,8]. Here, hydrogen peroxide, derived from superoxide by dismutation, takes center stage because of its particular chemical properties [9] as we will explain. Dismutation can be spontaneous, but more often, at low concentrations of superoxide, is mediated by superoxide dismutase-3 (SOD3), also known as extracellular SOD (EC-SOD).

In this review, we correlate the functionality and biology of NOX2 and SOD3 to delineate that these two enzymes, the oxidant producer and the dismutator, distribute and traffic in comparable ways to coordinate their activity at spatially confined microdomains of the cell surface. The result is a highly localized hydrogen peroxide production, which controls the activity of target proteins subject to redox control in the cytosol [10], at the cell surface [11], or even in neighboring cells [12].

## 2. Superoxide—The Initiator of Oxidative Capacity

**Figure 1:**
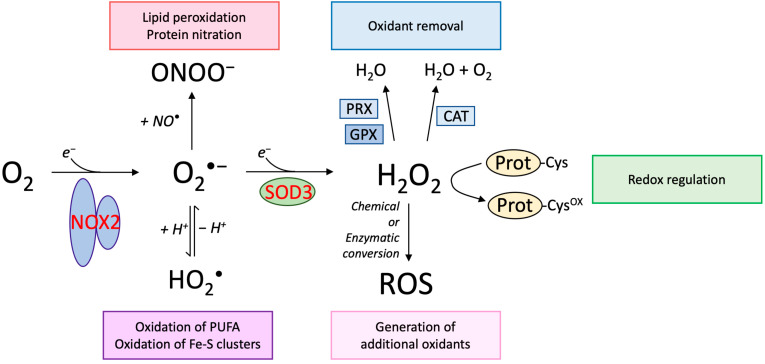
Biological impact of superoxide and hydrogen peroxide. The univalent reduction of molecular oxygen (O_2_) generates the superoxide radical (O_2_^•−^) and is enzymatically catalyzed by the NOX protein family, including NOX2. In biological systems at a physiological pH, superoxide is in equilibrium with the perhydroxyl radical, representing the protonated form of superoxide. Although the perhydroxyl radical only represents below 1% of superoxide at neutral pH, it is likely that this radical mediates some of the damaging impact of superoxide, indicated to include oxidation of polyunsaturated fatty acids (PUFA) and Fe-S clusters in proteins. Superoxide also participates in a very fast reaction with nitric oxide (NO^•^), which is also a radical. The reaction generates peroxynitrite (ONOO^−^), which supports oxidative reactions including lipid peroxidation and protein nitration. Hydrogen peroxide (H_2_O_2_) is generated by the univalent reduction of superoxide, a process that may proceed spontaneously or catalyzed by superoxide dismutase enzymes, including SOD3. Hydrogen peroxide is not a radical and express in general a low propensity to react with biomolecules. The oxidant may be removed by peroxiredoxins (PRX) and glutathione peroxidases (GPX), both expressed as distinct isoforms in cellular compartments. Moreover, catalase (CAT) present in peroxisomes is also capable of removing hydrogen peroxide. Specific redox-sensitive proteins display highly reactive cysteine residues (Prot-Cys) that may be oxidized by H_2_O_2_ to regulate biological activity (Prot-Cys^ox^), a process referred to as redox regulation. Hydrogen peroxide may also fuel the formation of other oxidants established by chemical or enzymatic conversion. See main text for further details.

The one-electron reduction of molecular oxygen generates superoxide (O_2_^•−^), which is a radical and an anion. This process is catalyzed by the NOX family of proteins, which includes NOX2 (Figure 1). As a redox-active species, superoxide may act as an oxidant, producing hydrogen peroxide or a reductant supporting the formation of molecular oxygen. Despite the connotation of being “super”, the radical is relatively unreactive [13]. Protonation of superoxide to form the perhydroxyl radical (HO_2_^•^) increases the reduction potential and hence reactivity, however, at physiological pH the pK*_a_*-value of the perhydroxyl radical (4.8) dictates that less than 1% of superoxide exists in this protonated form [14] (Figure 1). Nonetheless, it can be argued that despite the relative low levels, the perhydroxyl radical may represent the oxidizing capacity of superoxide [15]. Hence, HO_2_^•^ has been suggested to support the oxidation of polyunsaturated fatty acids [16] or enzymes encompassing Fe-S clusters [17,18]. Based on the relatively limited number of biological macromolecules subject to superoxide-mediated oxidation, it may be argued that the key role of superoxide in a biological context is to fuel the formation of other oxidants. Indeed, superoxide has the capacity of spontaneous dismutation to produce molecular oxygen and hydrogen peroxide. The spontaneous dismutation is supported by the formation of the perhydroxyl radical and hence fastest at low pH but presents a relatively high rate constant at physiological pH (k ~6 × 10^5^ M^−1^ s^−1^) [19]. Dismutation of superoxide is also catalyzed by superoxide dismutase enzymes, including SOD3 (Figure 1). Since the reaction of spontaneous dismutation is of second order, it is most prominent at relative high levels of superoxide, whereas at lower levels, the catalyzed first order reaction is likely to dominate [19]. In addition, superoxide reacts very fast and diffusion controlled with nitric oxide (NO^•^) to generate peroxynitrite (ONOO^−^). The rate constant of the reaction is at the top level of biological reactions (k ~10^10^ M^−1^ s^−1^) [20] and is 10.000× faster than the spontaneous dismutation, implying that superoxide will react with any NO^•^ generated in the immediate vicinity. Peroxynitrite is a strong oxidant and will support lipid peroxidation and protein nitrosylation, and therefore, may represent some of the oxidative damage induced by superoxide generation. In addition to supporting the formation of ONOO^−^, this fast reaction also removes bioactive NO^•^, which is well described to play a central role in vasodilation as well as in bone homeostasis [21]. Due to the charged state of superoxide it remains within topological barriers defined by biological membranes; the physiological relevance of channel-mediated superoxide transport (at high concentrations) across the plasma membrane is unclear [22]. 

## 3. Hydrogen Peroxide—The Messenger

Although superoxide has the capacity to generate hydrogen peroxide by spontaneous dismutation, a significant number of enzymes distributed in distinct subcellular compartments are known to produce hydrogen peroxide, including SOD3 [2]. At physiological conditions, the level of hydrogen peroxide in the intracellular space is regulated to ascertain a concentration of 1–100 nM, hundreds of times lower than extracellular concentration. Under these conditions of oxidative eustress, hydrogen peroxide acts as a first messenger to support the targeted oxidation of central cysteine residues of specific redox target proteins to regulate their biological function and activity, including cysteine residues of transcription factors and protein tyrosine phosphatases [2,10,23,24]. In light of this capacity, hydrogen peroxide is positioned as a key regulator of cellular responses. At persisting intracellular concentrations of hydrogen peroxide well above the baseline, redox signaling is severely hampered and hydrogen peroxide mediates the uncontrolled oxidation of biomolecules and disrupts biological activity, and consequently supports the development of oxidative distress [2]. In addition to oxidation-mediated effects, hydrogen peroxide fuels the generation of other oxidants by serving as a substrate for other enzymes [2,23].

The capacity of hydrogen peroxide to oxidize biological macromolecules is relatively low. However, the reactions with glutathione peroxidases (GPX) and peroxiredoxins (PRX) presents high rate constants [18], indicating that these enzymes are important in the removal of hydrogen peroxide to maintain a low concentration in cells and tissues [24]. As the reaction rates of hydrogen peroxide with these scavengers by far supersedes the reaction rates with most relevant redox-regulated target proteins, specific conditions must be met in order for biological redox-regulated events to occur [25,26]. Such conditions are established by the floodgate model, where the scavenging capacity of PRX is superseded and consequently allows hydrogen peroxide to mediate target-specific oxidation [27]. Reaction diffusion models of hydrogen peroxide signaling in human cells predict that hydrogen peroxide generated at the plasma membrane (as a bolus) achieves penetration distances and time scales of a few μm and 1 ms, respectively, before extinction by peroxiredoxin [28]. Although peroxiredoxins directly opposes hydrogen peroxide by consumption and thereby limits its penetration range into cytosol dramatically, the reductive capacity of cytosol is in fact required for the rapid return of hydrogen peroxide concentrations to baseline values in order to receive further signals [10]. Importantly, within the penetration distance a sufficient fraction of the redox protein population is oxidized to elicit a molecular signal, and it follows that any redistribution of redox target and oxidant source that will bring them in close proximity greatly increases efficiency of relay. If the redox target is localized outside of the effective penetration range (e.g., in the nucleus) the redox equivalents provided by hydrogen peroxide can also be transferred via a redox relay involving sensor proteins as, e.g., thioredoxin or protein disulfide isomerase [25]. Collectively, these arguments imply that autocrine redox signaling generally requires physical proximity between oxidant source and protein targets, and in the following we will discuss how sorting and membrane trafficking serves to bring together both the oxidant source as well as the oxidant modulator in order to mediate a biological signal.

## 4. NOX2 Is Contained in a Class of Agonist-Regulated Secretory Vesicles in Macrophages 

In general, NOX family members constitute the most important source of oxidants in cells [2]. Macrophages express both NOX1, and NOX2 [29,30], but we will concentrate here on NOX2 (the classical phagocyte NADPH Oxidase), which is by far the best described. NOX2 is composed of a membrane-bound flavocytochrome b_558_ complex, consisting of gp91phox and p22phox, and cytosolic subunits p40, p47, and p47phox in addition to Rac1/2. Only in response to cell stimulation will the cytosolic subunits traffic to cyt b_558_ in the membrane to assemble the holoenzyme and commence regulated superoxide production [31]. 

In neutrophils, the majority of NOX2 is contained within granules that are formed directly from the biosynthetic pathway. However, a population of NOX2 is also present in so-called secretory vesicles, which are formed by way of endocytosis (they also contain albumin) [32]. Human monocytes harbor a similar compartment containing complement receptor-3 (CR3) and NOX2 [33], but its origin or fate as monocytes differentiate to macrophages is unclear. In mature rodent bone marrow-derived macrophages and microglia, NOX2 is contained in an intracellular agonist-regulated storage compartment composed of numerous small (<100 nm) vesicles scattered in the cytosol [34]. The same compartment also seems present in human macrophages. As established by ultrastructural analysis and dynamic experimentation this NOX2-containing secretory compartment is clearly distinct from both biosynthetic and endocytic organelles, and presumably arises after endosomal sorting following clathrin-coated pit endocytosis of NOX2 [34]. Although interaction and retention factors for NOX2 on the cell surface are known [35], no sorting receptors for any NOX enzyme has been identified despite the presence of a hierarchy of undefined sorting signals [36,37]. More experimentation is clearly needed to understand the trafficking of NOX2 in mononuclear phagocytes. We speculate that NOX2 is contained in a bona fide agonist-regulated secretory compartment, and can undergo multiple cycles of exocytosis, endocytosis, and sorting akin to other known membrane proteins that contribute function to the cell surface for instance the GLUT4 transporter in adipocytes or aquaporin-2 in kidney duct cells [38]. Similar to these proteins, a minor proportion of the NOX2 population will constantly be in transit in early and recycling endosomes [39,40], but the final resting destination is the secretory vesicle compartment. 

The NOX2-containing vesicles are mobilized upon different inflammatory stimuli, e.g., tumor necrosis factor-α (TNFα) or CD40 ligand (CD40L), to enrich the cell surface in oxidant production, or they may be mobilized to phagosomes [34] often following homotypic fusion or fusion with endosomes as in neutrophils [34,41]. The small GTPases Rab27A/B is known to regulate mobility and exocytosis of so-called lysosome-related organelles in many cell types including hematopoietic cells [42,43,44]. It turns out that Rab27A/B are also crucial regulators of the trafficking and exocytosis of NOX2-containing compartments in neutrophils [42] and mononuclear phagocytes [34,45]. Both Rab27B [46] and Rab27A [44] interact directly with Munc13-4 to mediate SNAP/SNARE assembly for granule fusion with the plasma membrane. However, in neutrophils at least, Munc13-4 is not required for exocytosis of secretory vesicles [47]. An interesting study by Singh et al., indicates that in mast cells Rab27A may control mobility of granules by organization of the F-actin cytoskeleton and the association with myosin motor proteins, while Rab27B is the factor that mediates SNARE assembly and fusion [46]. The regulatory function of Rab27 in other cell types is likewise related to regulation of vesicular mobility through association with actin cytoskeleton [43,48].

In microglia, Rab27A and B expression seems to negatively regulate cell surface exposition of NOX2, in as much as Rab27A or B knock-down redistributes NOX2 from cell surface to the intracellular storage vesicles [34]. Rab27A similarly in mast cells negatively regulates granule exocytosis, likely by tethering of the granules to F-actin and regulating the integrity of the cortical F-actin cytoskeleton network [46], which functions as a barrier and blocks vesicle fusion with the plasma membrane. Depolymerization of cortical F-actin has long been known to increase the respiratory burst in different phagocyte cell types including microglia [49]. The phagosomal recruitment of NOX2 following immunoglobulin-opsonized prey (but nor complement-opsonized) is decreased as a consequence of either Rab27A or B knock down, indicating a positive regulatory role of Rab27 in this process [34] like in neutrophils [50]. In summary, Rab27A/B remains the best described regulator of NOX2 subcellular distribution in mononuclear phagocytes, but unknown sorting proteins and sorting mechanisms are bound to be operating in concert.

## 5. SOD3 Is Contained in Agonist-Regulated Vesicles in Macrophages

SOD3 was initially purified from human lung tissue and found to encompass structural and functional similarities to the intracellular isoenzyme SOD1, including the coordination of copper and zinc atoms as well as enzymatic activity supporting the dismutation of superoxide into hydrogen peroxide [51,52]. The protein is characterized by the presence of a C-terminal region involved in the binding to ligands in the extracellular space, including cell-surface proteoglycans via heparan sulfate and type I collagen [53,54,55]. Despite the presence of a signal peptide directing the protein to the secretory pathway, several studies have localized SOD3 to intracellular compartments in macrophages and neutrophils [56,57,58]. Detailed cellular characterization has shown, which SOD3 localizes to secretory vesicles of neutrophils [57], which also encompasses NOX2 [32]. Agonist stimulation of neutrophils supports the exocytosis of this cellular compartment, increasing the amount of SOD3 in both the extracellular space and, importantly, on the cell surface [57]. This release is accompanied by a reduced level of superoxide in the extracellular space and hence modulated redox conditions [57,59]. Macrophages can internalize recombinant SOD3 for storage, implicating a sorting step for deviation from the archetypical endosomal traffic to lysosomes [60]. In contrast to neutrophils that establish secretory vesicles by bulk membrane endocytosis, the uptake of SOD3 in macrophages appears to be mediated by the specific interaction with the cell surface receptor, low-density lipoprotein receptor-related protein 1 (LRP1), which is highly expressed in macrophages [60,61]. Agonist-induced release of SOD3 from macrophages increases both the level of the enzyme in the extracellular environment and on the cell surface within hours [60]. This release modulates the pro-inflammatory response of the macrophage by, e.g., reducing the level of released TNFα [60]. Hence, both neutrophils and macrophages encompass intracellular vesicles that readily can be mobilized to increase the level of SOD3 allowing for almost instantaneous and spatial modulation of the redox environment. 

## 6. SOD3 and NOX2 Interactions at the Macrophage Cell Surface, Redox Signaling

It is an appealing thought that NOX2 and SOD3 storage compartments may traffic to the macrophage cell surface together to unite function at this location in response to cellular stimulation. Similarly, trafficking of both vesicle populations to forming phagosomes might be functionally relevant, as hydrogen peroxide is the basis for bactericidal halide formation [23]. In that respect, macrophages differ from neutrophils where these two proteins are present within the same secretory vesicle population [57]. 

Yet another layer of spatial convergence of SOD3 and NOX2 is likely operating at the plasma membrane. NOX2 (like other NOX enzymes) is included in glycosphingolipid and cholesterol-enriched microdomains (lipid rafts) at the cell surface, which results in a focal oxidant production on the plasma membrane [62]. As mentioned, lipopolysaccharide (LPS) stimulation of macrophages causes the exocytosis and release of SOD3 [60], but remarkably, LPS also induces the translocation of cell surface-bound SOD3 from liquid-disordered membrane (general phospholipid environment) to lipid rafts [63,64]. This would ensure that both NOX2 and SOD3 are in close physical proximity in microdomains of the cell surface, which incidentally also serve as a recognized platform for receptor-mediated signaling. 

### 6.1. Autocrine Redox Signaling

In terms of autocrine cell signaling, hydrogen peroxide produced at high amplitude by the concerted action of NOX2 and SOD3 at the cell surface may either cross the plasma membrane to oxidize cytosolic target proteins [2,10] or directly react with cell surface resident receptors and other membrane proteins placed in the immediate vicinity to modulate their function and activity. This type of regulation is exemplified by integrins on the cell surface, which have been shown to interconvert between structures presenting high and low affinity. This interconversion is based on hydrogen peroxide-mediated oxidation of central cysteine residues [65,66]. In addition, several studies have suggested that ADAM17, which is responsible for shedding of a significant number of cell surface substrates, is subject to redox regulation [67,68]. The redox equivalents may not be provided directly by hydrogen peroxide but may be transferred by the action of cell-surface associated protein disulfide isomerase. These examples clearly show that there is a strong potential for regulating cell surface biology by utilizing redox regulation. Specifically, this allows for an immediate response to cellular stimulation (e.g., shedding of TNFα by ADAM17), potentiated by a plausible collaboration between NOX2 and SOD3.

**Figure 2:**
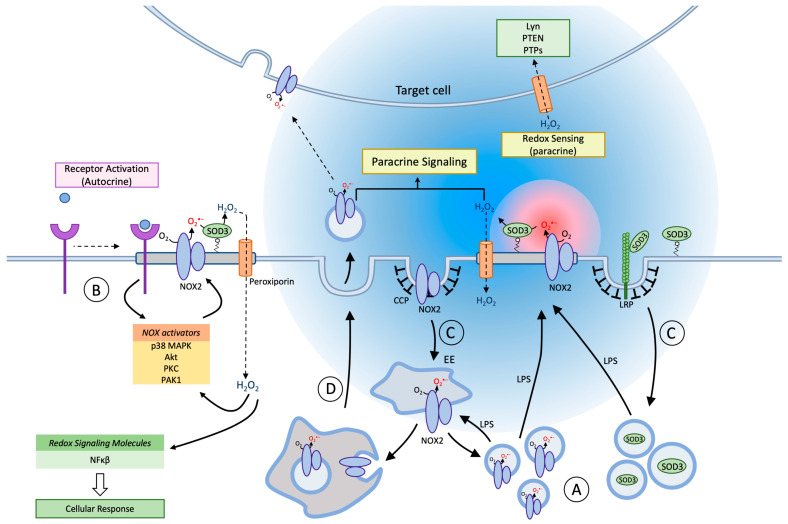
Membrane trafficking and Redox signaling of NOX2 and SOD3. In blood-derived macrophages and tissue macrophages, NOX2 and SOD3 are contained in separate agonist-responsive secretory vesicle populations distinct from, but communicating with, the endosomal compartment (**A**). Inflammatory activation (e.g., LPS) of macrophages causes mobilization and exocytosis of the vesicles to enrich the cell surface in superoxide production capacity (red sphere). On the cell surface NOX2 is organized in lipid rafts, and the agonist-induced planar inclusion of also SOD3 into this ensemble drives a rapid dismutation of superoxide to establish a steep gradient of hydrogen peroxide (blue sphere) for autocrine and paracrine signaling. Peroxiporin channels are likely also included in lipid rafts, which facilitates redox signaling by allowing hydrogen peroxide easier access to redox targets in the cytosol of the producing cell (autocrine) or in nearby cells (paracrine mode). Certain cell surface receptors signal directly or indirectly through redox circuits (**B**). Proximal signaling from the receptor causes activation of various NOX2 activators, and in the presence of SOD3 the ensuing hydrogen peroxide production amplifies, sustains and diversifies receptor signaling. NOX2-derived oxidants may also directly target a growing number of intracellular redox sensors e.g., nuclear factor kappa-light-chain-enhancer of activated B cells (Nf-kB) to induce altered signaling and transcription. Both NOX2 and SOD3 are internalized from the cell surface through clathrin-coated pits, and reach their respective storage compartments by unknown endosomal sorting mechanisms (**C**). A minor fraction of NOX2 will continuously be present in endosomal compartments and on the cell surface, but immune cell activation increases cell surface exposition of NOX2 and SOD3 several-fold by mobilization of secretory vesicles. The fraction of NOX2 in the endosomal pool is functionally important because NOX2, in an assembled and active format, can be sorted into intraluminal vesicles of multivesicular bodies/late endosomes, which are then discharged into the surroundings by exocytosis as exosomes (**D**). Exosomes can now confer novel oxidant production capacity to neighboring target cells, after fusion with their plasma or endosomal membranes to insert the NOX2 complex.

In macrophages, a battery of sensing innate immune cell receptors are all coupled to NOX2 activation [69]. Receptor occupancy induces proximal signaling, which activates the cytosolic phox subunits to translocate to cyt b_558_ in the membrane and commence superoxide production. Superoxide produced at the cell surface is then dismutated via SOD3 activity to hydrogen peroxide, which is able to enter cells either through diffusion through the membrane, or through select aquaporins (called peroxiporins [70]) that allow hydrogen peroxide to travel down its concentration gradient into the cortical cytosol where is reacts with protein targets (Figure 2; left). Redox signaling may then reinforce or diversify signaling from the cell surface receptor or directly activate signaling pathways or gene transcription programs [8,10]. Intriguingly, peroxiporins themselves may also be lipid raft-resident proteins [71,72,73], and colocalization with NOX2 in the same class of lipid raft is probable given the co-immunoprecipitation of NOX2 with peroxiporins [74]. Therefore, the entire ensemble of signaling receptor, oxidant producer, oxidant modulator, and transporter may be confined within lipid rafts for optimal signaling [26]. 

Monocytes depend on NOX-derived oxidants for differentiation into macrophages [30], and the acquisition of specific macrophage activation states or metabolic profiles is to a large extent also under redox control [7,75]. Macrophages may polarize to either an M1 phenotype (proinflammatory, pathogen elimination, oxidant production including NO) or an M2 phenotype (chemokine expression, increased phagolysosomal capacity, resolution of inflammation) that is instilled in response to local cues in tissues. The term polarization indicates that a spectrum of phenotypes exists in between the M1 and M2 states [76]. Stimuli that induce the M1 phenotype increase NOX2 activity and concomitant oxidant production, while the M2 state is correlated with a dramatically reduced transcription of NOX genes and lower oxidant output (see refs. in [7]). The phenotype assumed has important pathological corollaries in chronic diseases such as neurodegeneration or cancer. For example, the tumor environment-induced transition to an M2 phenotype of tumor-associated macrophages is beneficial for tumor growth and metastasis, and experimental therapeutic regimes that would inhibit acquisition of the M2, or convert it to an M1 phenotype, are currently being investigated [77]. While, it is clear that NOX-derived oxidants govern macrophage differentiation and polarization the specifics have not been hammered out yet. In vivo, both microglia and macrophages have been reported to depend on NOX2 activation for acquisition of the M1 phenotype following inflammatory stimuli [78,79]. In fact, in p47phox-deficient mice, microglia exposed to stimuli that normally induce M1 polarization, instead assume an M2 phenotype [79]. However, reports since then have indicated that also acquisition of the M2 activation state involves NOX2 and/or NOX1 derived oxidants in murine macrophages in vitro or in vivo [30,80]. The former study also calls into question the role of NOX1/NOX2 in shaping the M1 phenotype. This is worth keeping in mind, as there is a growing consensus that NOX2 is required for the limitation of the inflammatory response by several mechanisms [81]; genetic or experimental deficiency of NOX2 in man and rodents, respectively, causes hyperinflammatory conditions and autoimmunity [7,82]. It is interesting to note that in microglia, interleukin-1β (IL-1β) secretion following LPS stimulation is a result of NOX1-mediated signaling, which localizes to late endolysosomal compartments [29]. Because of the very limited penetration ranges of hydrogen peroxide it is entirely conceivable that more than one redox signaling pathway can be in operation at the same time, as long as the distance between oxidant sources exceeds the penetration distance.

To what extent SOD3 is necessary for autocrine redox signaling has not been addressed in detail, but in macrophages the increased presence of SOD3 on the cell surface following exocytosis of intracellular storage vesicles decreases release of TNFα when compared to cells lacking SOD3 [60]. The capacity to regulate the inflammatory response correlates with the known function of NOX2 to limit excess production of inflammatory mediators [7,82]. Looking to other cell types, the association of SOD3 with lipid rafts has been reported in endothelial cells to regulate the hydrogen peroxide-mediated activity of protein tyrosine phosphatases (PTPs) in the proximate microenvironment and thereby increase vascular endothelial growth factor (VEGFR) signaling exclusively in the lipid rafts [63]. In a separate study investigating the impact of SOD3 on receptor tyrosine kinase-signaling, the positive impact of SOD3 in cellular proliferation, was found to be mediated by an increased activation of the RAS protein involved in the Ras-Erk signaling cascade [83]. Interestingly, the SOD3-induced activation of RAS was abrogated by the presence of the NOX inhibitor DPI, indicating that the effect of SOD3 on RAS signaling is supported by NOX activity. Notably, the level of SOD3 expression may have opposite effects indicating that cellular proliferation is indeed regulated by a delicate redox balance in part supported by NOX and SOD3 activities [84] (and references therein).

### 6.2. Paracrine Redox Communication

Interestingly, the low reactivity of hydrogen peroxide towards biological macromolecules in general, allows the molecule to diffuse on the scale of several hundred micrometers up to perhaps one mm in the extracellular space where reductive capacity is low [18,85]. While the omnipresent antioxidant defense precludes intercellular communication over longer distances, e.g., between tissues and organs [2], there are recognized instances where hydrogen peroxide acts as a direct intercellular first messenger within tissues (Figure 2; right) [12].

B- and T-lymphocytes undergo profound changes in metabolism upon activation, and both basal and activation-induced metabolism is shaped by redox signaling pathways [75]. Antigen presenting cells or regulatory lymphocyte subsets often form very close cell surface associations with either B- or T-cells to regulate their function. The prime example of such close contact is the immunological synapse between a macrophage and a T-lymphocyte in the process of antigen presentation. Using rodent models of autoimmune disease, the group of Rikard Holmdahl has thoroughly documented that deficiency of NOX2 activity specifically in macrophages causes hyperinflammatory conditions and autoimmunity due to hyper activation of T-lymphocytes [86,87]. By genetic dissection, it has been possible to establish that macrophage oxidant production via NOX2 modulates the T-cell surface redox conditions to induce anergy or tolerance [88,89]. In humans the association between full or partial NOX2 deficiency and autoimmunity is more complex [23]. The very close proximity of macrophage and T-cell in the immunological synapse makes the structure an ideal physical platform for intercellular redox signaling. In that respect, it could be speculated that SOD3 has a role in this system. The ability of SOD3 to attenuate inflammation has previously been reported in relation to a range of inflammatory conditions [90]. Adenoviral delivery of SOD3 into arthritic foot pads of rats does indeed decrease inflammation, however, the effect seems to be independent of the antioxidant function of SOD3 [91], and the role if any of SOD3 in the immunological synapse is uncertain. It may be that the distances involved are sufficiently small that rapid dismutation of superoxide to hydrogen peroxide is not required. Other examples of paracrine redox communication in mammals have been proposed but not proven [92,93,94]. The central nervous system potentially poses an environment where paracrine redox communication would have optimal conditions for function as the interstitial space is very limited and distances between cells is small. It has been proposed that microglia via NOX2-derived oxidants induce long term synaptic depression in neurons following hypoxia and LPS stimulation [92], but the source of oxidants was not verified genetically, and the redox targets remain unknown. Because CR3 ligation invariably results in superoxide production by NOX2, it will be interesting to see whether paracrine redox signaling is involved in the microglial stripping of surplus synapses that takes place through CR3 [95]. The caudal fin lesion model of the Zebrafish has provided interesting insight into oxidants role in tissue repair [12]. Following a lesion of the caudal fin, a tissue scale gradient of oxidant production builds up, which attracts leukocytes [96] and supports the regeneration of sensory axons lesioned simultaneously with the primary injury or even as a separate axotomy event some distance away [97]. In this scenario, wounded keratinocytes produce hydrogen peroxide [97], which in turn attracts leukocytes via direct activation of the small tyrosine kinase Lyn [96], to coordinate/promote axon regeneration [97]. While zebrafish broadly express other NOX enzymes including NOX2, it is very telling that the oxidant producer in this wound model system is DUOX expressed by keratinocytes [98]. As DUOX is equipped with a peroxidase-like domain the oxidant produced is hydrogen peroxide, and there is no need for extracellular SOD activity (zebrafish SOD3 binds with low affinity to the cell surface; [99]). The identification in leukocytes of Lyn as a sensor of the extracellular redox environment is an important step in the delineation of the molecular changes that are instigated in target cells mediated by an extracellular redox signal [96]. More work along this avenue will likely yield valuable novel information [100]. The demonstration of paracrine signaling, particularly in living tissues, is technically very demanding because oxidant source, extracellular diffusion, and oxidant targets all have to be taken into account. To the extent that these parameters are not gauged with very stringent measures (cell type-specific genetic control of oxidant source, scavenging of extracellular oxidants, and determination of redox targets in recipient cells) many studies have provided good evidence for [12], but not directly proven, paracrine redox signaling. In many studies, a loop of autocrine redox-stimulated release of paracrine soluble mediators could equally well account for the observations [101].

#### NOX2 in Exosomes

**Figure 3:**
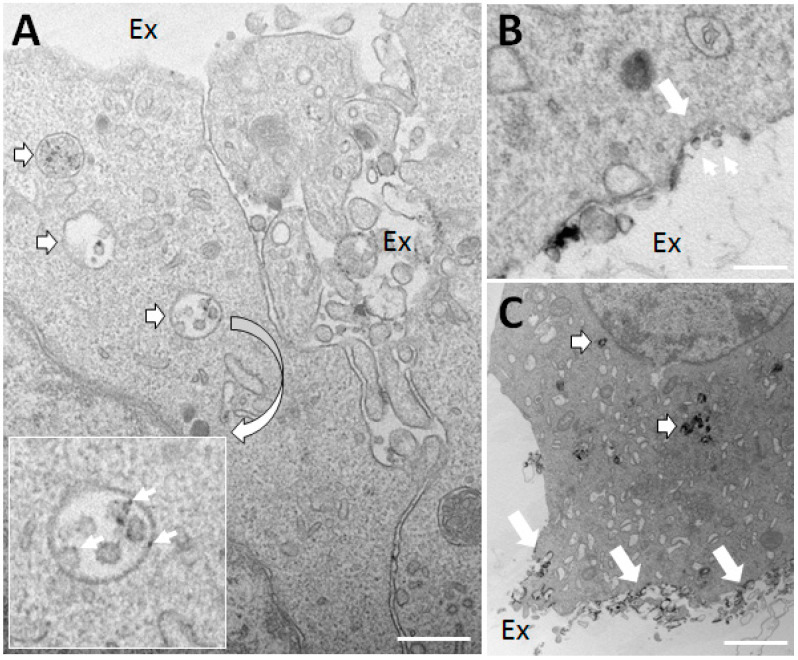
PC12 nerve cells in the process of differentiation secrete exosomes with oxidant production capacity. (**A**,**B**) PC12 neuron-like cells under differentiation induced by nerve growth factor (NGF) for four days were treated with phorbol myristate acetate (PMA) for one hour (to activate NADPH oxidase) in the presence of cerium chloride (CeCl_3_), a cytochemical agent, which forms an electron-dense precipitate upon reaction with hydrogen peroxide. (**A**) Note the presence of multivesicular bodies/late endosomes (outlined white arrows) containing intraluminal vesicles (exosomes to be) with clear deposition of CeCl_3_ (see inset) indicative of hydrogen peroxide production. Ex, extracellular space. (**B**) A profile suggesting the exocytosis of a late endosome (large white arrow) leading to the release of exosomes with hydrogen peroxide production (small arrows). (**C**) In addition to activating NOX2, PMA is a strong secretagogue. After three hours of PMA stimulation a countable number of late endosomes are clearly stained with CeCl_3_ precipitates (outlined white arrows), and substantial amounts of material with precipitate is seen deposited on the cell surface following their exocytosis (large white arrows). Bars, 250 nm in (**A**,**B**); 2 μm in (**C**).

Recently, another surprising layer of intercellular redox communication has been uncovered. Two recent articles demonstrate the transfer of exosomes containing NOX2 and oxidant production from one cell type to another (Figure 2; center). The observed transfer effectuates an altered physiological response in the target cells including suppression of T-helper lymphocyte proliferation [102] or regeneration of injured axons [103]. Exosomes are formed when the limiting membrane of multivesicular bodies/late endosomes buds inwards and pinches off to generate intraluminal vesicles of approximately 50–100 nm in size. Subsequently, when late endosomes fuse with the plasma membrane in an exocytic event, the vesicles are released to the surroundings and are now by convention called exosomes [104]. Normally, regulatory CD8+ T-cells (T_regs_) suppress CD4+ T-cell activation in secondary immune tissues, but this mechanism fails (in part) in aging individuals. In the work of Wen et al., the authors demonstrate that this deficit is caused by down-regulation of NOX2 in T_regs_, and can be countered by reconstitution of NOX2 expression levels [102]. Remarkably, T_regs_ exert their suppressive effect on CD4+ T-cells by the exosomal transfer of preassembled NOX2 to the CD4+ T-cells. A tight interface reminiscent of the immunological synapse, is formed between the two cell types. Whether, exosomal transfer of NOX2 also occurs between macrophages and CD4+ T-cells in the process of antigen stimulation [105] has to our knowledge not been addressed. A second study shows that local macrophages are instrumental in the regenerative response in the injured sciatic nerve and dorsal root ganglia [103]. Their effect is mediated by the exosomal transfer of an active NOX2 complex directly to injured axons. Following exosome endocytosis and fusion with neuron late endomembranes, NOX2 travels retrogradely to reach the soma area. At this location, NOX2-produced oxidants inactivates the phosphatase PTEN to activate the PI3K–Akt pathway and regenerative outgrowth. We recently used cerium chloride (CeCl_3_)-based cytochemical techniques to detect oxidant production in living cells at ultrastructural resolution for the unequivocal determination of oxidant production in the exosomes themselves (See Figure 3). Exosomal transport of NOX2 is therefore not exclusive to immune cells.

The studies mentioned above are very well performed and together with the observations illustrated in Figure 3 open up for a delightful black box of molecular uncertainty on several levels. First of all, NOX2 must be assumed subject to regulated sorting into late endosomal intraluminal vesicles by an unknown sorting mechanism, which would also include cytosolic phox proteins and Rac1 (i.e., the ensemble would be activated upon sorting). Curiously, the released exosomes are capable of oxidant production, which is striking since it is generally accepted that a constant cycling of the phox proteins from the cytosolic pool to cyt b_558_ in the membrane is required for sustained superoxide production [106]. Moreover, it is unclear how NADPH is provided in the interior of the exosome.

Additionally, the work of Hervera et al., forwards the possibility that NOX2 transferred to neuronal endosomes from macrophage exosomes, carries out its redox signaling from late endosomes in the soma area after retrograde transport [103]. The concept of endosomal redox signaling is appealing, because it would offer a mechanism for the directed translocation of an oxidant source to its oxidant targets (rather than the other way around), which in a large and polarized cell type such as a neuron could prove particularly important. However, as NOX enzymes are electrogenic (electrons from NADPH are transferred across the membrane), oxidant production would quickly cease in the absence of a charge neutralizing mechanism. Mechanistic evidence for endosomal redox signaling has for now only been presented by two different groups [107,108], one of which proposes the H^+^/Cl^−^ exchange transporter 3 (ClC-3) as a charge neutralizer in endosomes of neutrophils [109]. In the intervening roughly ten years since the original publications, no additional evidence by any group has been put forward to confirm, substantiate, or further explore this interesting area.

No experimental evidence for the inclusion of SOD3 into exosomes exist, but recent findings show that mesenchymal stem cells release extracellular vesicles encompassing SOD3, and importantly that these vesicles have the capacity to modulate the redox conditions of recipient cells [110,111]. Although no direct corporation between NOX2 and SOD3 has been reported, it is intriguing to speculate that exosomes may encompass both enzymes to produce hydrogen peroxide at the target cell. This plausible setting will extend the mode of action for hydrogen peroxide as an intercellular messenger.

## 7. Concluding Remarks

The prefix anti- in antioxidant is a word-forming element of Greek origin meaning “against, opposed to, opposite of, instead”. While antioxidants certainly can make oxidants go away, it is also clear that they have important accessory roles in shaping distinct cellular microenvironments where they orchestrate the level and type of oxidants produced and consequently support redox relays that transmit cellular ques to relevant target proteins. Specifically, the activity of SOD enzymes may both be referred to as antioxidants in their capacity to remove superoxide but also as a prooxidant in providing hydrogen peroxide. Understanding how different cells, including macrophages, dynamically redistribute their batteries of oxidant producers, modulators, and antioxidants upon cellular stimulation will require more research into molecular sorting motifs and membrane trafficking pathways that govern the spatial organization of these elements, including that of NOX2 and SOD3. Many biological responses are dependent on autocrine redox signaling, and the multitude of intracellular redox targets now constitute the “redoxome”. With offset in future research, it will in particular be interesting to realize to what extent cell surface proteins constitute meaningful targets for rapid hydrogen peroxide-mediated regulation of activity. As better and more sensitive biologically encoded sensors are continuously developed it will no doubt spur on the elucidation of direct paracrine redox signaling in living tissues as well. The concept of paracrine redox signaling has now been expanded by the revelation of macrophage exosomes as vehicles of NOX2 oxidant production, a phenomenon that could prove to be wide-spread in the different tissues of our body. The notion in the present review is that the expanding appreciation of cellular processes subject to redox regulation dictates that we must aim to further describe the intricate network of oxidants and antioxidants that supports the generation of distinct redox microenvironments. Specifically, we argue that the subcellular distribution of NOX2 and SOD3 in inflammatory cells supports the formation of such microenvironments.

## References

[1] Holmstrom K.M., Finkel T. (2014). Cellular mechanisms and physiological consequences of redox-dependent signalling. Nat. Rev. Mol. Cell Biol..

[2] Sies H., Jones D.P. (2020). Reactive oxygen species (ROS) as pleiotropic physiological signalling agents. Nat. Rev. Mol. Cell Biol..

[3] Bedard K., Krause K.H. (2007). The NOX family of ROS-generating NADPH oxidases: Physiology and pathophysiology. Physiol. Rev..

[4] Lambeth J.D. (2004). NOX enzymes and the biology of reactive oxygen. Nat. Rev. Immunol..

[5] Segal A.W., Jones O.T. (1978). Novel cytochrome b system in phagocytic vacuoles of human granulocytes. Nature.

[6] Pizzolla A., Hultqvist M., Nilson B., Grimm M.J., Eneljung T., Jonsson I.M., Verdrengh M., Kelkka T., Gjertsson I., Segal B.H. (2012). Reactive oxygen species produced by the NADPH oxidase 2 complex in monocytes protect mice from bacterial infections. J. Immunol..

[7] Vilhardt F., Haslund-Vinding J., Jaquet V., McBean G. (2016). Microglia antioxidant systems and redox signaling. Br. J. Pharmacol..

[8] Rojo A.I., McBean G., Cindric M., Egea J., Lopez M.G., Rada P., Zarkovic N., Cuadrado A. (2014). Redox control of microglial function: Molecular mechanisms and functional significance. Antioxid. Redox Signal..

[9] Sies H. (2014). Role of metabolic H2O2 generation: Redox signaling and oxidative stress. J. Biol. Chem..

[10] Marinho H.S., Real C., Cyrne L., Soares H., Antunes F. (2014). Hydrogen peroxide sensing, signaling and regulation of transcription factors. Redox Biol..

[11] Metcalfe C., Cresswell P., Ciaccia L., Thomas B., Barclay A.N. (2011). Labile disulfide bonds are common at the leucocyte cell surface. Open Biol..

[12] Hervera A., Santos C.X., De Virgiliis F., Shah A.M., Di Giovanni S. (2019). Paracrine Mechanisms of Redox Signalling for Postmitotic Cell and Tissue Regeneration. Trends Cell Biol..

[13] Collin F. (2019). Chemical Basis of Reactive Oxygen Species Reactivity and Involvement in Neurodegenerative Diseases. Int. J. Mol. Sci..

[14] McCord J.M., Fridovich I. (1969). Superoxide dismutase. An enzymic function for erythrocuprein (hemocuprein). J. Biol. Chem..

[15] De Grey A.D. (2002). HO2*: The forgotten radical. DNA Cell Biol..

[16] Panov A. (2018). Perhydroxyl Radical (HO2^(*)) as Inducer of the Isoprostane Lipid Peroxidation in Mitochondria. Mol. Biol..

[17] Flint D.H., Tuminello J.F., Emptage M.H. (1993). The inactivation of Fe-S cluster containing hydro-lyases by superoxide. J. Biol. Chem..

[18] Winterbourn C.C. (2008). Reconciling the chemistry and biology of reactive oxygen species. Nat. Chem. Biol..

[19] Sheng Y., Abreu I.A., Cabelli D.E., Maroney M.J., Miller A.F., Teixeira M., Valentine J.S. (2014). Superoxide dismutases and superoxide reductases. Chem. Rev..

[20] Ferrer-Sueta G., Radi R. (2009). Chemical biology of peroxynitrite: Kinetics, diffusion, and radicals. ACS Chem. Biol..

[21] Klein-Nulend J., van Oers R.F., Bakker A.D., Bacabac R.G. (2014). Nitric oxide signaling in mechanical adaptation of bone. Osteoporos. Int..

[22] Hawkins B.J., Madesh M., Kirkpatrick C.J., Fisher A.B. (2007). Superoxide flux in endothelial cells via the chloride channel-3 mediates intracellular signaling. Mol. Biol. Cell.

[23] Nauseef W.M. (2019). The phagocyte NOX2 NADPH oxidase in microbial killing and cell signaling. Curr. Opin. Immunol..

[24] Winterbourn C.C. (2018). Biological Production, Detection, and Fate of Hydrogen Peroxide. Antioxid. Redox Signal..

[25] Winterbourn C.C. (2020). Hydrogen peroxide reactivity and specificity in thiol-based cell signalling. Biochem. Soc. Trans..

[26] Nordzieke D.E., Medraño-Fernandez I. (2018). The Plasma Membrane: A Platform for Intra- and Intercellular Redox Signaling. Antioxidants.

[27] Wood Z.A., Poole L.B., Karplus P.A. (2003). Peroxiredoxin evolution and the regulation of hydrogen peroxide signaling. Science.

[28] Lim J.B., Langford T.F., Huang B.K., Deen W.M., Sikes H.D. (2016). A reaction-diffusion model of cytosolic hydrogen peroxide. Free Radic. Biol. Med..

[29] Cheret C., Gervais A., Lelli A., Colin C., Amar L., Ravassard P., Mallet J., Cumano A., Krause K.H., Mallat M. (2008). Neurotoxic activation of microglia is promoted by a nox1-dependent NADPH oxidase. J. Neurosci..

[30] Xu Q., Choksi S., Qu J., Jang J., Choe M., Banfi B., Engelhardt J.F., Liu Z.G. (2016). NADPH Oxidases Are Essential for Macrophage Differentiation. J. Biol. Chem..

[31] Vignais P.V. (2002). The superoxide-generating NADPH oxidase: Structural aspects and activation mechanism. Cell. Mol. Life Sci..

[32] Sengelov H., Nielsen M.H., Borregaard N. (1992). Separation of human neutrophil plasma membrane from intracellular vesicles containing alkaline phosphatase and NADPH oxidase activity by free flow electrophoresis. J. Biol. Chem..

[33] Calafat J., Kuijpers T.W., Janssen H., Borregaard N., Verhoeven A.J., Roos D. (1993). Evidence for small intracellular vesicles in human blood phagocytes containing cytochrome b558 and the adhesion molecule CD11b/CD18. Blood.

[34] Ejlerskov P., Christensen D.P., Beyaie D., Burritt J.B., Paclet M.H., Gorlach A., van Deurs B., Vilhardt F. (2012). NADPH Oxidase is Internalized by Clathrin-Coated Pits and Localizes to a Rab27A/B GTPase-Regulated Secretory Compartment in Activated Macrophages. J. Biol. Chem..

[35] Ikeda S., Yamaoka-Tojo M., Hilenski L., Patrushev N.A., Anwar G.M., Quinn M.T., Ushio-Fukai M. (2005). IQGAP1 regulates reactive oxygen species-dependent endothelial cell migration through interacting with Nox2. Arter. Thromb. Vasc. Biol..

[36] Helmcke I., Heumüller S., Tikkanen R., Schröder K., Brandes R.P. (2009). Identification of structural elements in Nox1 and Nox4 controlling localization and activity. Antioxid. Redox Signal..

[37] von Lohneysen K., Noack D., Wood M.R., Friedman J.S., Knaus U.G. (2010). Structural insights into Nox4 and Nox2: Motifs involved in function and cellular localization. Mol. Cell. Biol..

[38] Chieregatti E., Meldolesi J. (2005). Regulated exocytosis: New organelles for non-secretory purposes. Nat. Rev. Mol. Cell Biol..

[39] Casbon A.J., Allen L.A., Dunn K.W., Dinauer M.C. (2009). Macrophage NADPH oxidase flavocytochrome B localizes to the plasma membrane and Rab11-positive recycling endosomes. J. Immunol..

[40] Johnson J.L., He J., Ramadass M., Pestonjamasp K., Kiosses W.B., Zhang J., Catz S.D. (2016). Munc13-4 Is a Rab11-binding Protein That Regulates Rab11-positive Vesicle Trafficking and Docking at the Plasma Membrane. J. Biol. Chem..

[41] Kobayashi T., Robinson J.M., Seguchi H. (1998). Identification of intracellular sites of superoxide production in stimulated neutrophils. J. Cell Sci..

[42] Johnson J.L., Brzezinska A.A., Tolmachova T., Munafo D.B., Ellis B.A., Seabra M.C., Hong H., Catz S.D. (2010). Rab27a and Rab27b Regulate Neutrophil Azurophilic Granule Exocytosis and NADPH oxidase Activity by Independent Mechanisms. Traffic.

[43] Mizuno K., Tolmachova T., Ushakov D.S., Romao M., Abrink M., Ferenczi M.A., Raposo G., Seabra M.C. (2007). Rab27b regulates mast cell granule dynamics and secretion. Traffic.

[44] Neeft M., Wieffer M., de Jong A.S., Negroiu G., Metz C.H., van Loon A., Griffith J., Krijgsveld J., Wulffraat N., Koch H. (2005). Munc13-4 is an effector of rab27a and controls secretion of lysosomes in hematopoietic cells. Mol. Biol. Cell.

[45] Jancic C., Savina A., Wasmeier C., Tolmachova T., El-Benna J., Dang P.M., Pascolo S., Gougerot-Pocidalo M.A., Raposo G., Seabra M.C. (2007). Rab27a regulates phagosomal pH and NADPH oxidase recruitment to dendritic cell phagosomes. Nat. Cell Biol..

[46] Singh R.K., Mizuno K., Wasmeier C., Wavre-Shapton S.T., Recchi C., Catz S.D., Futter C., Tolmachova T., Hume A.N., Seabra M.C. (2013). Distinct and opposing roles for Rab27a/Mlph/MyoVa and Rab27b/Munc13-4 in mast cell secretion. FEBS J..

[47] Brzezinska A.A., Johnson J.L., Munafo D.B., Crozat K., Beutler B., Kiosses W.B., Ellis B.A., Catz S.D. (2008). The Rab27a effectors JFC1/Slp1 and Munc13-4 regulate exocytosis of neutrophil granules. Traffic.

[48] Desnos C., Schonn J.S., Huet S., Tran V.S., El-Amraoui A., Raposo G., Fanget I., Chapuis C., Menasche G., de Saint Basile G. (2003). Rab27A and its effector MyRIP link secretory granules to F-actin and control their motion towards release sites. J. Cell Biol..

[49] Rasmussen I., Pedersen L.H., Byg L., Suzuki K., Sumimoto H., Vilhardt F. (2010). Effects of F/G-actin ratio and actin turn-over rate on NADPH oxidase activity in microglia. BMC Immunol..

[50] Anderson K.E., Chessa T.A., Davidson K., Henderson R.B., Walker S., Tolmachova T., Grys K., Rausch O., Seabra M., Tybulewicz V.L. (2011). PtdIns3P and Rac direct the assembly of the NADPH oxidase on a novel, pre-phagosomal compartment during FcR-mediated phagocytosis in primary mouse neutrophils. Blood.

[51] Hjalmarsson K., Marklund S.L., Engström A., Edlund T. (1987). Isolation and sequence of complementary DNA encoding human extracellular superoxide dismutase. Proc. Nat. Acad. Sci. USA.

[52] Marklund S.L. (1982). Human copper-containing superoxide dismutase of high molecular weight. Proc. Nat. Acad. Sci. USA.

[53] Karlsson K., Lindahl U., Marklund S.L. (1988). Binding of human extracellular superoxide dismutase C to sulphated glycosaminoglycans. Biochem. J..

[54] Petersen S.V., Oury T.D., Ostergaard L., Valnickova Z., Wegrzyn J., Thogersen I.B., Jacobsen C., Bowler R.P., Fattman C.L., Crapo J.D. (2004). Extracellular superoxide dismutase (EC-SOD) binds to type i collagen and protects against oxidative fragmentation. J. Biol. Chem..

[55] Adachi T., Marklund S.L. (1989). Interactions between human extracellular superoxide dismutase C and sulfated polysaccharides. J. Biol. Chem..

[56] Manni M.L., Tomai L.P., Norris C.A., Thomas L.M., Kelley E.E., Salter R.D., Crapo J.D., Chang L.Y., Watkins S.C., Piganelli J.D. (2011). Extracellular superoxide dismutase in macrophages augments bacterial killing by promoting phagocytosis. Am. J. Pathol..

[57] Iversen M.B., Gottfredsen R.H., Larsen U.G., Enghild J.J., Praetorius J., Borregaard N., Petersen S.V. (2016). Extracellular superoxide dismutase is present in secretory vesicles of human neutrophils and released upon stimulation. Free Radic. Biol. Med..

[58] Loenders B., Van Mechelen E., Nicolaï S., Buyssens N., Van Osselaer N., Jorens P.G., Willems J., Herman A.G., Slegers H. (1998). Localization of extracellular superoxide dismutase in rat lung: Neutrophils and macrophages as carriers of the enzyme. Free Radic. Biol. Med..

[59] Break T.J., Witter A.R., Indramohan M., Mummert M.E., Dory L., Berg R.E. (2016). Extracellular Superoxide Dismutase Enhances Recruitment of Immature Neutrophils to the Liver. Infect. Immun..

[60] Hu L., Zachariae E.D., Larsen U.G., Vilhardt F., Petersen S.V. (2019). The dynamic uptake and release of SOD3 from intracellular stores in macrophages modulates the inflammatory response. Redox Biol..

[61] Petersen S.V., Thøgersen I.B., Valnickova Z., Nielsen M.S., Petersen J.S., Poulsen E.T., Jacobsen C., Oury T.D., Moestrup S.K., Crapo J.D. (2010). The concentration of extracellular superoxide dismutase in plasma is maintained by LRP-mediated endocytosis. Free Radic. Biol. Med..

[62] Vilhardt F., van Deurs B. (2004). The phagocyte NADPH oxidase depends on cholesterol-enriched membrane microdomains for assembly. EMBO J..

[63] Oshikawa J., Urao N., Kim H.W., Kaplan N., Razvi M., McKinney R., Poole L.B., Fukai T., Ushio-Fukai M. (2010). Extracellular SOD-derived H2O2 promotes VEGF signaling in caveolae/lipid rafts and post-ischemic angiogenesis in mice. PLoS ONE.

[64] Gottfredsen R.H., Goldstrohm D.A., Hartney J.M., Larsen U.G., Bowler R.P., Petersen S.V. (2014). The cellular distribution of extracellular superoxide dismutase in macrophages is altered by cellular activation but unaffected by the naturally occurring R213G substitution. Free Radic. Biol. Med..

[65] Yan B., Smith J.W. (2000). A redox site involved in integrin activation. J. Biol. Chem..

[66] Bergerhausen L., Grosche J., Meißner J., Hecker C., Caliandro M.F., Westerhausen C., Kamenac A., Rezaei M., Mörgelin M., Poschmann G. (2020). Extracellular Redox Regulation of α7β Integrin-Mediated Cell Migration Is Signaled via a Dominant Thiol-Switch. Antioxidants.

[67] Willems S.H., Tape C.J., Stanley P.L., Taylor N.A., Mills I.G., Neal D.E., McCafferty J., Murphy G. (2010). Thiol isomerases negatively regulate the cellular shedding activity of ADAM17. Biochem. J..

[68] Düsterhöft S., Jung S., Hung C.W., Tholey A., Sönnichsen F.D., Grötzinger J., Lorenzen I. (2013). Membrane-proximal domain of a disintegrin and metalloprotease-17 represents the putative molecular switch of its shedding activity operated by protein-disulfide isomerase. J. Am. Chem. Soc..

[69] Haslund-Vinding J., McBean G., Jaquet V., Vilhardt F. (2016). NADPH oxidases in Microglia oxidant production: Activating Receptors, Pharmacology, and Association with Disease. Br. J. Pharmacol..

[70] Bienert G.P., Chaumont F. (2014). Aquaporin-facilitated transmembrane diffusion of hydrogen peroxide. Biochim. Biophys. Acta.

[71] Zheng X., Bollinger Bollag W. (2003). Aquaporin 3 colocates with phospholipase d2 in caveolin-rich membrane microdomains and is downregulated upon keratinocyte differentiation. J. Investig. Dermatol..

[72] Mazzone A., Tietz P., Jefferson J., Pagano R., LaRusso N.F. (2006). Isolation and characterization of lipid microdomains from apical and basolateral plasma membranes of rat hepatocytes. Hepatology.

[73] Ishikawa Y., Yuan Z., Inoue N., Skowronski M.T., Nakae Y., Shono M., Cho G., Yasui M., Agre P., Nielsen S. (2005). Identification of AQP5 in lipid rafts and its translocation to apical membranes by activation of M3 mAChRs in interlobular ducts of rat parotid gland. Am. J. Physiol. Cell Physiol..

[74] Hara-Chikuma M., Satooka H., Watanabe S., Honda T., Miyachi Y., Watanabe T., Verkman A.S. (2015). Aquaporin-3-mediated hydrogen peroxide transport is required for NF-kappaB signalling in keratinocytes and development of psoriasis. Nat. Commun..

[75] Muri J., Kopf M. (2020). Redox regulation of immunometabolism. Nat. Rev. Immunol..

[76] Martinez F.O., Gordon S. (2014). The M1 and M2 paradigm of macrophage activation: Time for reassessment. F1000prime Rep..

[77] Brown J.M., Recht L., Strober S. (2017). The Promise of Targeting Macrophages in Cancer Therapy. Clin. Cancer Res..

[78] Padgett L.E., Burg A.R., Lei W., Tse H.M. (2015). Loss of NADPH oxidase-derived superoxide skews macrophage phenotypes to delay type 1 diabetes. Diabetes.

[79] Choi S.H., Aid S., Kim H.W., Jackson S.H., Bosetti F. (2012). Inhibition of NADPH oxidase promotes alternative and anti-inflammatory microglial activation during neuroinflammation. J. Neurochem..

[80] Griess B., Mir S., Datta K., Teoh-Fitzgerald M. (2020). Scavenging reactive oxygen species selectively inhibits M2 macrophage polarization and their pro-tumorigenic function in part, via Stat3 suppression. Free Radic. Biol. Med..

[81] Trevelin S.C., Dos Santos C.X., Ferreira R.G., de Sá Lima L., Silva R.L., Scavone C., Curi R., Alves-Filho J.C., Cunha T.M., Roxo-Júnior P. (2016). Apocynin and Nox2 regulate NF-κB by modifying thioredoxin-1 redox-state. Sci. Rep..

[82] Deffert C., Carnesecchi S., Yuan H., Rougemont A.L., Kelkka T., Holmdahl R., Krause K.H., Schappi M.G. (2012). Hyperinflammation of chronic granulomatous disease is abolished by NOX2 reconstitution in macrophages and dendritic cells. J. Pathol..

[83] Laurila J.P., Castellone M.D., Curcio A., Laatikainen L.E., Haaparanta-Solin M., Gronroos T.J., Marjamaki P., Martikainen S., Santoro M., Laukkanen M.O. (2009). Extracellular superoxide dismutase is a growth regulatory mediator of tissue injury recovery. Mol. Ther..

[84] Parascandolo A., Laukkanen M.O. (2019). Carcinogenesis and Reactive Oxygen Species Signaling: Interaction of the NADPH Oxidase NOX1-5 and Superoxide Dismutase 1-3 Signal Transduction Pathways. Antioxid. Redox Signal..

[85] Jelcic M., Enyedi B., Xavier J.B., Niethammer P. (2017). Image-Based Measurement of H(2)O(2) Reaction-Diffusion in Wounded Zebrafish Larvae. Biophys. J..

[86] Olofsson P., Holmberg J., Tordsson J., Lu S., Akerstrom B., Holmdahl R. (2003). Positional identification of Ncf1 as a gene that regulates arthritis severity in rats. Nat. Genet..

[87] Hultqvist M., Olofsson P., Holmberg J., Backstrom B.T., Tordsson J., Holmdahl R. (2004). Enhanced autoimmunity, arthritis, and encephalomyelitis in mice with a reduced oxidative burst due to a mutation in the Ncf1 gene. Proc. Nat. Acad. Sci. USA.

[88] Gelderman K.A., Hultqvist M., Holmberg J., Olofsson P., Holmdahl R. (2006). T cell surface redox levels determine T cell reactivity and arthritis susceptibility. Proc. Nat. Acad. Sci. USA.

[89] Gelderman K.A., Hultqvist M., Pizzolla A., Zhao M., Nandakumar K.S., Mattsson R., Holmdahl R. (2007). Macrophages suppress T cell responses and arthritis development in mice by producing reactive oxygen species. J. Clin. Investig..

[90] Nguyen N.H., Tran G.B., Nguyen C.T. (2020). Anti-oxidative effects of superoxide dismutase 3 on inflammatory diseases. J. Mol. Med..

[91] Kelkka T., Laurila J.P., Sareila O., Olofsson P., Laukkanen M.O., Holmdahl R. (2012). Superoxide dismutase 3 limits collagen-induced arthritis in the absence of phagocyte oxidative burst. Mediat. Inflamm..

[92] Zhang J., Malik A., Choi H.B., Ko R.W., Dissing-Olesen L., MacVicar B.A. (2014). Microglial CR3 activation triggers long-term synaptic depression in the hippocampus via NADPH oxidase. Neuron.

[93] Cascino T., Csanyi G., Al Ghouleh I., Montezano A.C., Touyz R.M., Haurani M.J., Pagano P.J. (2011). Adventitia-derived hydrogen peroxide impairs relaxation of the rat carotid artery via smooth muscle cell p38 mitogen-activated protein kinase. Antioxid. Redox Signal..

[94] Al Ghouleh I., Frazziano G., Rodriguez A.I., Csanyi G., Maniar S., St Croix C.M., Kelley E.E., Egana L.A., Song G.J., Bisello A. (2013). Aquaporin 1, Nox1, and Ask1 mediate oxidant-induced smooth muscle cell hypertrophy. Cardiovasc. Res..

[95] Schafer D.P., Lehrman E.K., Kautzman A.G., Koyama R., Mardinly A.R., Yamasaki R., Ransohoff R.M., Greenberg M.E., Barres B.A., Stevens B. (2012). Microglia sculpt postnatal neural circuits in an activity and complement-dependent manner. Neuron.

[96] Yoo S.K., Starnes T.W., Deng Q., Huttenlocher A. (2011). Lyn is a redox sensor that mediates leukocyte wound attraction in vivo. Nature.

[97] Rieger S., Sagasti A. (2011). Hydrogen peroxide promotes injury-induced peripheral sensory axon regeneration in the zebrafish skin. PLoS Biol..

[98] Niethammer P., Grabher C., Look A.T., Mitchison T.J. (2009). A tissue-scale gradient of hydrogen peroxide mediates rapid wound detection in zebrafish. Nature.

[99] Matthiesen C.L., Hua L., Torsleva A.S., Poulsen E.T., Larsen U.G., Kjaer-Sorensen K., Thomsen J.S., Brüel A., Enghild J.J., Oxvig C. (2021). Superoxide dismutase 3 is expressed in bone tissue and required for normal bone homeostasis and mineralization. Free Radic. Biol. Med..

[100] Wu W., Hale A.J., Lemeer S., den Hertog J. (2017). Differential oxidation of protein-tyrosine phosphatases during zebrafish caudal fin regeneration. Sci. Rep..

[101] Amblard I., Thauvin M., Rampon C., Queguiner I., Pak V.V., Belousov V., Prochiantz A., Volovitch M., Joliot A., Vriz S. (2020). H(2)O(2) and Engrailed 2 paracrine activity synergize to shape the zebrafish optic tectum. Commun. Biol..

[102] Wen Z., Shimojima Y., Shirai T., Li Y., Ju J., Yang Z., Tian L., Goronzy J.J., Weyand C.M. (2016). NADPH oxidase deficiency underlies dysfunction of aged CD8+ Tregs. J. Clin. Investig..

[103] Hervera A., De Virgiliis F., Palmisano I., Zhou L., Tantardini E., Kong G., Hutson T., Danzi M.C., Perry R.B., Santos C.X.C. (2018). Reactive oxygen species regulate axonal regeneration through the release of exosomal NADPH oxidase 2 complexes into injured axons. Nat. Cell Biol..

[104] Pegtel D.M., Gould S.J. (2019). Exosomes. Annu. Rev. Biochem..

[105] Holmdahl R., Sareila O., Pizzolla A., Winter S., Hagert C., Jaakkola N., Kelkka T., Olsson L.M., Wing K., Backdahl L. (2013). Hydrogen peroxide as an immunological transmitter regulating autoreactive T cells. Antioxid. Redox Signal..

[106] Li X.J., Tian W., Stull N.D., Grinstein S., Atkinson S., Dinauer M.C. (2009). A fluorescently tagged C-terminal fragment of p47phox detects NADPH oxidase dynamics during phagocytosis. Mol. Biol. Cell.

[107] Lamb F.S., Hook J.S., Hilkin B.M., Huber J.N., Volk A.P., Moreland J.G. (2012). Endotoxin priming of neutrophils requires endocytosis and NADPH oxidase-dependent endosomal reactive oxygen species. J. Biol. Chem..

[108] Oakley F.D., Smith R.L., Engelhardt J.F. (2009). Lipid rafts and caveolin-1 coordinate interleukin-1beta (IL-1beta)-dependent activation of NFkappaB by controlling endocytosis of Nox2 and IL-1beta receptor 1 from the plasma membrane. J. Biol. Chem..

[109] Moreland J.G., Davis A.P., Matsuda J.J., Hook J.S., Bailey G., Nauseef W.M., Lamb F.S. (2007). Endotoxin priming of neutrophils requires NADPH oxidase-generated oxidants and is regulated by the anion transporter ClC-3. J. Biol. Chem..

[110] Yang J.W., Seo Y., Shin T.H., Ahn J.S., Oh S.J., Shin Y.Y., Kang M.J., Lee B.C., Lee S., Kang K.S. (2020). Extracellular Vesicles from SOD3-Transduced Stem Cells Exhibit Improved Immunomodulatory Abilities in the Murine Dermatitis Model. Antioxidants.

[111] Khanh V.C., Yamashita T., Ohneda K., Tokunaga C., Kato H., Osaka M., Hiramatsu Y., Ohneda O. (2020). Rejuvenation of mesenchymal stem cells by extracellular vesicles inhibits the elevation of reactive oxygen species. Sci. Rep..

